# Supplementary material for: Polyphenol and flavonoid content in major Teff [Eragrostis tef (Zuccagni) Trotter] varieties in Amhara Region, Ethiopia
Source: PLoS One. 2022 Aug 2;17(8):e0272010. doi: 10.1371/journal.pone.0272010 (PMC9345355; doi:10.1371/journal.pone.0272010)
Supplement: Attachment — Submitted filename: Review_2.docx [file pone.0272010.s002.docx]

Ref. No.: PONE-D-22-08028

Title: Polyphenol and flavonoid content in major Teff [Eragrostis tef (Zuccagni) Trotter]

varieties in Amhara Region, Ethiopia

Authors responded to comments by significantly improving the quality of the manuscript. In my opinion, the work in this formcan be accepted for its publication in Plos One.
