# Supplementary material for: Polyphenol and flavonoid content in major Teff [Eragrostis tef (Zuccagni) Trotter] varieties in Amhara Region, Ethiopia
Source: PLoS One. 2022 Aug 2;17(8):e0272010. doi: 10.1371/journal.pone.0272010 (PMC9345355; doi:10.1371/journal.pone.0272010)
Supplement: Attachment — Submitted filename: Response to the reviewer comments.docx [file pone.0272010.s003.docx]

**Replies to the technical comments of the reviewers and Editors**

Ref. No.: PONE-D-22-08028

**Title: Polyphenol and flavonoid content in major Teff [Eragrostis tef (Zuccagni) Trotter]**

**varieties in Amhara Region, Ethiopia**

We are very thankful to the reviewers and Editors for their appreciation of our manuscript entitled **Polyphenol and flavonoid content in major Teff [*Eragrostis tef (Zuccagni) Trotte*r]varieties in Amhara Region, Ethiopia**, and the constructive criticism on certain issues. The replies to the Reviewers' and editor's comments are given carefully below:

**Response to reviewer #1 comments.**

**Comments #1**:  Some little typing and punctuation errors were still observed (e.g. in the tile and in the test lacking spaces)

**Response #1:** Significant typographic errors and punctuation corrections have been taken throughout the manuscript.

**Comments #2:**  In the Introduction replace "It's a chasmogamous C4 annual cereal with a fibrous…" with "Teff is a chasmogamous C4 annual…";

**Response #2:** The reviewer's suggestion is incorporated in the revised manuscript.

**Comment #3:** In the Results and Discussion, Figure 1 on gallic acid results is Figure 2;

Response # 3: Figure 1 refers to a map of the sampling areas, while Figure 2 refers to the gallic acid standard's calibration curve and UV spectra.

**Comment #4:**  In the Results and Discussion, I suggest to modify the following sentence "This implies that brown or mixed teff variety (from Awi zone) contained a higher total phenolic content than Kuncho teff variety (from East Gojjam and West Gojjam zone) and Tseday teff variety (from Awi zone)." Some samples from the West Gojjam zone have a high TPC, indeed the mean value of TPC and TFC-Q.E were found to be the highest in West Gojjam teff samples compared to the samples from other zones

**Response #4:** The brown or mixed teff variety from Awi zone contained a higher total phenolic content than the white teff (Tseday variety ) collected from the Awi zone. The mean TPC values were compared between the two white teff varieties (Kuncho and Tseday) collected from the three administrative zones. It was noted that the Kuncho teff variety collected from the West Gojjam Zone contained a higher mean TPC value than the same Kuncho variety collected from the East Gojjam zone and the other variety (Tsedey) from the Awi zone.

**Comment #5:** Among varieties from the Awi zone, the brown samples showed the highest TPC. Similar comments could be also arranged for the TFC.

**Response #5:** Thank you once again for pointing out this. We have modified the phrase as follow: Similar to the TPC value, the brown (mixed) teff samples from the Awi zone had higher flavonoid contents (Table 1) than the white variety from this sampling zone. Comparing the mean value of TFC between the three sampling zones, samples from the west Gojjam zone had a mean TFC-Q.E value higher than samples from East Gojjam and Awi zones

**Comment #6**: In the Results and Discussion, in the sentence "On the other hand, relatively comparable TPC values with the present study were reported by [14] for white and brown teff varieties collected from the Tigray region of Ethiopia." could be interest to specify the values reported by the reference authors

**Response #6:** Thank you for your comments. We have now incorporated the data reported by [14} as follows. On the other hand, 263 mg GAE/100g and 448 mg GAE /100g, respectively, for white (Tseday type) and brown teff varieties collected from the Tigray region of Ethiopia were reported by [14].

**Comment #7:** Table 3 must be insert in the text as a Figure or as a Table?

**Response #7**: We are grateful for this comment. We have changed table 3 into Figure 6. Then the previous figure 6 is labeled as figure 7, and the last figure 7 is captioned figure 8 in the revised manuscript. This change has been corrected in the text too.
